# Expression of selected regulatory molecules on the CD83+ monocyte-derived dendritic cells generated from patients with laryngeal cancer and their clinical significance

**DOI:** 10.1007/s00405-013-2510-4

**Published:** 2013-04-30

**Authors:** Janusz Klatka, Ewelina Grywalska, Maria Klatka, Magdalena Wasiak, Adrian Andrzejczak, Jacek Rolinski

**Affiliations:** 1 Department of Otolaryngology and Laryngeal Oncology, Medical University of Lublin, Jaczewskiego 8 Street, 20-954 Lublin, Poland; 2 Department of Clinical Immunology and Immunotherapy, Medical University of Lublin, Chodzki 4a Street, 20-093 Lublin, Poland; 3 Department of Pediatric Endocrinology and Diabetology, Medical University of Lublin, Chodzki 2 Street, 20-093 Lublin, Poland

**Keywords:** B7H1, B7H4, CD200, CD200R, Monocyte-derived dendritic cells, Cell lysates, Laryngeal cancer

## Abstract

B7H1 and B7H4 overexpression is associated with inhibition of the immune system in many solid tumors, and altogether with CD200 molecule plays an important role in tumor invasion by promoting malignant transformation. However, there is no report about impact of these molecules on laryngeal squamous cell carcinoma. The objective of the present study was to assess by means of flow cytometry the expression of B7H1, B7H4, CD200, and CD200R on CD83+ monocyte-derived dendritic cells (Mo-DC), pulsed with autologous tumor cell lysates (aTCL) in patients who suffer from G1, G2, or G3 laryngeal carcinoma (LC, *n* = 60) in comparison to healthy donors (HD, *n* = 15). It has been demonstrated that median value of the percentages of CD83+ B7H1+, CD83+ B7H4+, and CD83+ CD200+ cells were higher in LC patients than HD (*p* = 0.041, *p* ≤ 0.0001, and *p* = 0.02, respectively). Mean fluorescence intensity (MFI) of CD200, CD200R, B7H1, and B7H4 on the Mo-DC pulsed with aTCL of the patients was also higher than on the Mo-DC of HD (*p* ≤ 0.0001, *p* ≤ 0.0001, *p* = 0.002, and *p* ≤ 0.0001, respectively). The highest MFI levels of all molecules were noted in grade 3 LC. The aforementioned results prove that there is a relation between the presence of laryngeal cancer and the expression of B7H1, B7H4, CD200, and CD200R regulatory molecules on the CD83+ Mo-DC pulsed with autologous cancer cell lysates. Strong association of LC grade and the tested antigens expression suggests a critical role for these proteins in LC biology.

## Introduction

Laryngeal cancer is one of the most commonly occurring malicious cancers of head and neck region, with squamous cell carcinoma as the predominant histologic type [1, 2]. In spite of their constant development, the classical therapeutic methods, such as surgical procedures or radiotherapy are not sufficiently effective in prolonging the survival time of cancer patients and improving the quality of their lives [3]. Therefore, the increasing morbidity and unsatisfactory effects of treatment, especially in cases of advanced laryngeal carcinoma (LC), stimulate the researchers to develop new more effective therapy schemes including immunotherapy with the use of the dendritic cells (DC) and/or monoclonal antibodies [4]. Unfortunately, an optimal method of treatment with DC has not been developed yet due to the limited knowledge of the biology of DC, the influence of cancer antigens on their functions, and the expression of co-stimulatory molecules which are extremely vital as far as the interaction of DC with other cells is concerned [5].

B7H1 and B7H4 molecules belong to newly discovered proteins from B7 family. B7H1 molecule demonstrates hindering properties toward the immunological response of T lymphocytes [6]. B7H4 molecule was identified in 2003 as a co-stimulatory molecule which is vital in the process of hindering the proliferation of T lymphocytes and blocking the production of cytokines by Th1 and Th2 subpopulations. Its high expression on the surface of cells of some cancers may lead to an assumption that it is a marker of carcinogenesis [7]. CD200 (formerly OX2) is a type 1a membrane protein with two extracellular immunoglobulin superfamily domains, a single transmembrane region, and a short cytoplasmic tail [8, 9], broadly expressed on a variety of cell types, such as some DC, thymocytes, T and B lymphocytes, neurons, kidney glomeruli, syncytiotrophoblasts, and endothelial cells [10], which delivers immunoregulatory signals through binding to receptors expressed on monocytes/myeloid cells, including monocyte-derived dendritic cells (Mo-DC), and T lymphocytes. Signals delivered through the CD200:CD200R axis have been shown to play an important role in the regulation of anti-tumor immunity. Overexpression of CD200 has been reported in a number of malignancies and on cancer stem cells [11] and this molecule may play roles in local tumor invasion as well as augments the metastatic capacity of squamous cell carcinoma [12].

The main aim of the present study was to assess the expression of B7H1, B7H4, CD200 and CD200R on CD83+ Mo-DC, pulsed with autologous tumor cell lysates (aTCL), of patients who suffer from LC in comparison to healthy donors (HD). To the best of our knowledge, the present publication is the first to describe the mentioned issue. Thus far, the expression of the aforementioned molecules has not been assessed yet on DC in patients with LC. The analysis seems to be essential for many reasons—it will allow us to determine whether it is possible to derive fully efficient DC from patients with LC, which respond to cancer antigens, and what is the influence of LC antigens on Mo-DC. We have also assessed the impact of LC in various histological grades on the expression of B7H1, B7H4, CD200, and CD200R molecules.

## Materials and methods

### Patients and healthy controls

Sixty male patients treated surgically for primarily diagnosed squamous cell carcinoma of the larynx, without preoperative treatment, were included in the study. The diagnosis of squamous cell carcinoma of the larynx was established by histopathology of tumor samples. The mean age of patients was 64.72 ± 9.29 years (ranging from 45 to 79 years; median 65.8).

Peripheral blood from 15 healthy male donors, at the mean age of 57.68 ± 11.95 (ranging from 43 to 73 years; median 59), was used as a control. In patients and HDs, peripheral blood WBC was within the normal range between 4 and 10 G/L.

None of the patients and controls had signs of infection at the time of investigation and for a month before surgery none had been taking drugs of known influence on the immune system. None of the patients or HDs had undergone blood transfusion. Persons with allergic diseases in anamnesis were excluded from the study. The research protocol was approved by the local Ethics Committee and all patients gave written informed consent.

### Isolation of the peripheral blood mononuclear cells

Peripheral blood mononuclear cells (PBMCs) were separated from heparinized venous blood of the patients and the HDs by density gradient centrifugation using Gradisol-L (Aqua Medica, Poland) and centrifuged for 20 min at 700 G. PBMCs were collected and washed twice in phosphate-buffered saline (PBS) without Ca2+ or Mg2+ (Biochrom AG, Germany). Interphase cells were washed twice in PBS without Ca2+ or Mg2+ (Biochrom AG, Germany) and then resuspended at 0.5–1 × 10^6^ cells. Their viability was checked by Trypan Blue (Sigma-Aldrich, Germany) staining in light microscopy.

### Preparation of the neoplastic cell lysates

Laryngeal cancer tissue was obtained during surgical treatment. Tumor samples (without necrotic areas) were digested with 1 mg/ml of type I collagenase (Biochrome AG, Germany), 1 mg/ml of type I deoxyribonucleic (Sigma, Germany), and 0.1 mg/ml of hyaluronidase (Sigma, Germany) and washed twice in RPMI 1640 (Biochrome, Germany). Tumor cells were homogenized by five repeated cycles of quick freezing (−80 °C) and thawing (37 °C). Large particles were removed by centrifugation and after that supernatants were passed through a 20 μm pore filter.

### Dendritic cells generation

Peripheral blood mononuclear cells were incubated with anti-CD14 microbeads (Miltenyi-Biotec, Bergisch Gladbach, Germany) and passed through MACS separation columns according to the manufacturer’s instructions. The CD14+ cell population was used to prepare Mo-DC. After isolation, the CD14+ cells were washed twice with PBS and seeded into cell culture flasks. The CD14+ cells were cultured in RPMI 1640 medium (BioWhittaker, Walkersville, MD, USA) supplemented with 2 % human albumin (Baxter, Austria), 100 IU/ml penicillin, 50 mg/ml streptomycin, and 100 mg/ml neomycin (Sigma-Aldrich, Germany) for 2 h in 37 °C and 5 % CO_2_. After incubation, the non-adherent cells were removed with PBS without Ca^2+^ and Mg^2+^ (Biochrom AG, Germany). Adherent cells were grown in the appropriate culture medium for 7 days. On days 1, 3, and 5 of the culture, 1,000 IU/ml rhGM-CSF (Leukine, Berlex, USA) and 500 IU/ml rhIL-4 (Miltenyi-Biotec, Bergisch Gladbach, Germany) were added. The cells were then divided into two equal parts and the first part was assessed using flow cytometry method without stimulation with tumor cell lysates. Tumor necrosis factor α (rhTNF-α, Strathmann, Germany) at a concentration of 50 ng/ml and the aTCL were added to the second part during the last 48 h of culture for the maturation and pulsing of the Mo-DC.

The Mo-DC of healthy controls were generated as above but were not pulsed with aTCL due to the absence of cancer tissue in this group. Generated cells were then harvested using Trypsin/EDTA solution (Biochrome, Germany).

### Flow cytometric analysis

We used the followings monoclonal antibodies (mAb): anti-CD83 FITC (BD Pharmingen, USA)/anti-B7H1 PE (Biolegend, USA), anti-CD83 FITC (BD Pharmingen, USA)/anti-B7H4 PE (Biolegend, USA), anti-CD83 FITC (BD Bioscience, USA)/anti-CD200 PE (AbD Serotec, USA), and anti-CD83 FITC (BD Bioscience, USA)/anti-CD200R PE (AbD Serotec, USA). The cell phenotype characterization was performed using a FACSCalibur cytometer equipped with 488 nm argon laser (Becton–Dickinson, USA) and analyzed with CellQuest Software (Becton–Dickinson, USA). We collected 300,000 of events in total. Cell debris and dead cells were excluded from the analysis based on scatter signal.

### Statistical analysis

Statistica 9 PL (Stat Soft Inc.) software was applied to statistical analysis and Mann–Whitney *U* test was used. Pearson’s linear correlation coefficient (*r*) was calculated to disclose relationships between variables. All results are showed as mean ± standard deviation (SD), medians, and minimum and maximum values. *p* value <0.05 was considered statistically significant.

## Results

The results are presented in the form of percentage values of the cells with the expression of a given antigen and the mean fluorescence intensity (MFI), which is the mean score of the density of an expression of a given molecule on a cell. Table 1 presents obtained results in G1, G2, G3 LC patients, as in HD.

**Table 1:** Percentages of CD83+, CD83+ CD200+, CD83+ CD200R+, CD83+ B7H1+, and CD83+ B7H4+ Mo-DC generated from laryngeal cancer patients in various histological grades (G1–G3) and from healthy donors, and mean fluorescence intensity (MFI) of analyzed molecules on Mo-DC

	Minimum	Maximum	Mean	Median	Standard deviation
Laryngeal cancer grade 1 (G1), *n* = 15 patients					
CD83+ cells (%)	71.45	99.22	90.14	95.75	9.45
CD83 MFI	231.16	506.69	323.43	306.30	87.70
CD83+ CD200+ cells (%)	8.82	41.28	16.42	13.55	8.19
CD200 MFI	41.56	153.82	66.75	50.32	40.67
CD83+ CD200R+ cells (%)	29.03	87.27	67.92	74.87	18.41
CD200R MFI	188.63	451.68	274.16	242.47	88.16
CD83+ B7H1+ cells (%)	42.13	92.01	58.31	48.48	18.86
B7H1 MFI	78.84	281.38	154.47	164.92	70.93
CD83+ B7H4+ cells (%)	1.14	4.02	2.11	1.83	1.01
MFI B7H4	11.44	52.88	24.59	19.58	14.20
Laryngeal cancer grade 2 (G2), *n* = 23 patients					
CD83+ cells (%)	55.37	99.67	89.67	94.72	10.85
CD83 MFI	201.23	628.26	327.35	268.68	129.43
CD83+ CD200+ cells (%)	6.17	44.47	16.51	13.12	9.96
CD200 MFI	49.8	166.45	95.97	108.22	38.68
CD83+ CD200R+ cells (%)	45.93	95.34	76.28	79.49	14.46
CD200R MFI	239.42	504.09	318.91	289.80	78.04
CD83+ B7H1+ cells (%)	47.94	94.83	80.89	83.68	12.12
B7H1 MFI	153.08	335.72	214.97	201.77	43.60
CD83+ B7H4 +cells (%)	1.77	4.69	3.16	3.16	0.72
MFI B7H4	19.58	57.92	38.86	39.58	10.37
Laryngeal cancer grade 3 (G3), *n* = 22 patients					
CD83+ cells (%)	69.27	94.49	83.52	83.79	8.26
CD83 MFI	211.23	626.29	356.33	327.94	118.77
CD83+ CD200+ cells (%)	6.17	42.47	15.17	13.95	8.82
CD200 MFI	52.97	415.09	184.09	172.44	102.56
CD83+ CD200R+ cells (%)	33.75	94.01	70.32	82.15	18.17
CD200R MFI	254.34	874.18	603.04	549.01	214.64
CD83+ B7H1+ cells (%)	61.83	100.28	91.86	95.71	10.67
B7H1 MFI	175.03	623.68	415.05	424.93	155.81
CD83+ B7H4+ cells (%)	2.26	16.16	6.82	5.38	4.41
MFI B7H4	23.43	90.31	63.08	63.02	20.54
Healthy donors, *n* = 15 persons					
CD83+ cells (%)	99.41	99.86	99.73	99.77	0.14
CD83 MFI	139.72	276.38	199.23	192.00	40.28
CD83+ CD200+ cells (%)	6.12	22.68	11.22	10.79	3.85
CD200 MFI	14.41	24.47	18.16	18.25	2.83
CD83+ CD200R+ cells (%)	36.21	89.05	66.78	65.87	12.38
CD200R MFI	16.99	124.78	81.62	86.73	24.93
CD83+ B7H1+ cells (%)	42.95	94.72	71.27	74.66	15.70
B7H1 MFI	102.93	261.15	164.83	163.82	46.08
CD83+ B7H4+ cells (%)	0.47	5.97	1.96	1.82	1.34
MFI B7H4	1.23	7.43	4.11	4.02	1.69

**Fig. 1:**
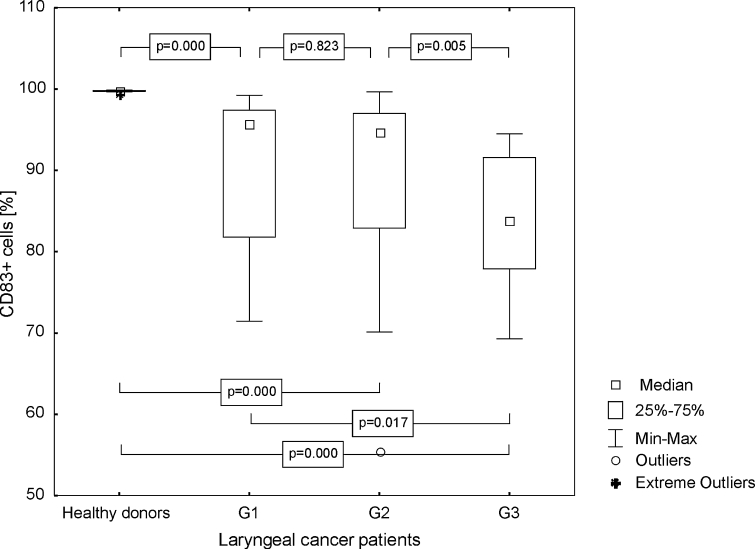
Assessment of the frequencies of CD83+ Mo-DC in control group and laryngeal cancer patients with grade 1, grade 2, or grade 3 squamous cells carcinoma of the larynx

**Fig. 2:**
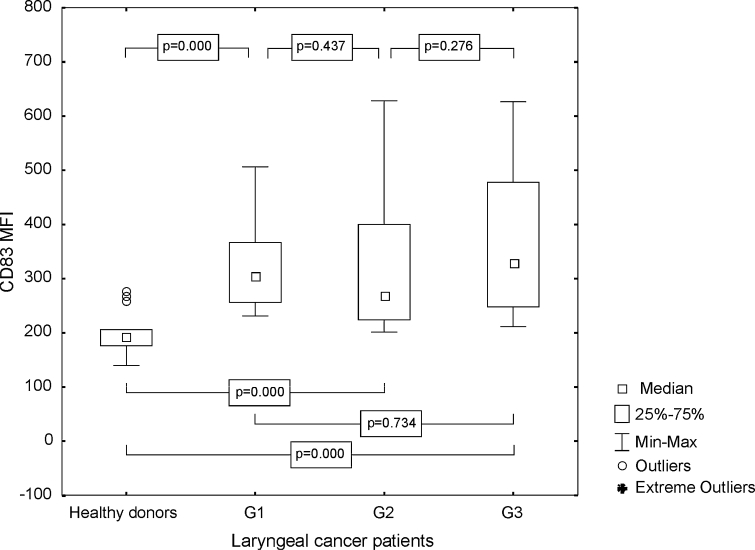
Assessment of the CD83 MFI values on Mo-DC in control group and laryngeal cancer patients with grade 1, grade 2, or grade 3 squamous cells carcinoma of the larynx

Our study revealed that the percentage of Mo-DC with an expression of CD83 antigen in patients was statistically lower than in HDs (median 91.28 %, range from 55.37 to 99.67 %; mean 87.53 ± 9.94 vs. 99.77 %, range from 99.41 to 99.86 %; mean 99.73 ± 0.14 %, *p* ≤ 0.0001). There was no difference between the percentages of Mo-DC generated from patients with G1 and G2 LC (*p* = 0.823). Lower frequencies of CD83+ Mo-DC were noted in patients with G3 LC in comparison to G1 LC (*p* = 0.017). Significantly higher percentages of CD83+ Mo-DC were generated from HD than from G1, G2, and G3 LC patients (*p* ≤ 0.0001), and from G2 than G3 LC patients (*p* = 0.005). Figure 1 illustrates observed results. The differences of CD83 MFI values between the aforementioned groups were also statistically significant (*p* ≤ 0.0001); higher in patients (median 308.44, range from 201.23 to 628.26; mean 336.99 ± 115.39) than in healthy individuals (median 192.01, range from 139.72 to 276.38; mean 199.22 ± 40.28). Figure 2 presents obtained results.

**Fig. 3:**
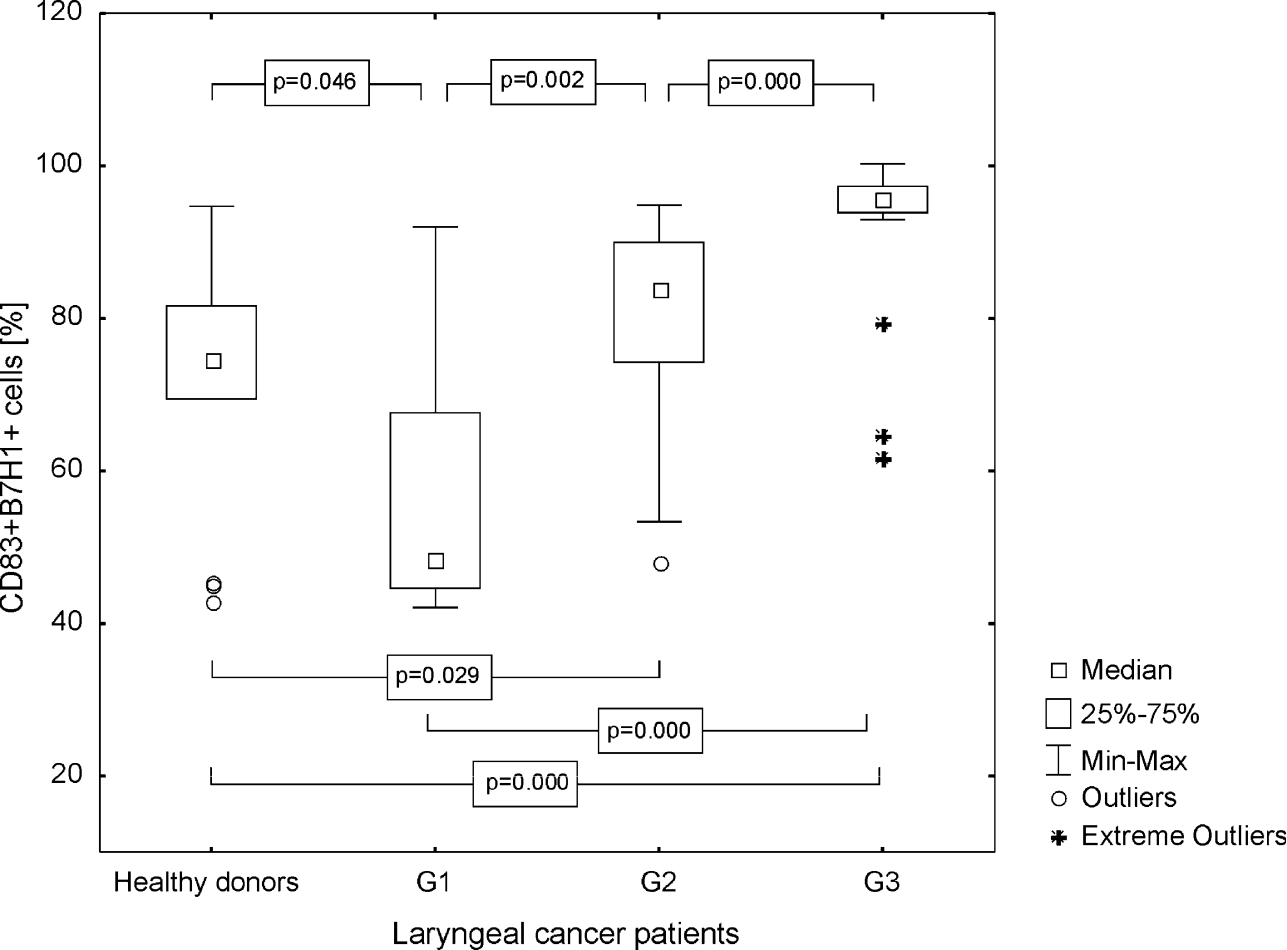
Assessment of the frequencies of CD83+ B7H1+ Mo-DC in control group and laryngeal cancer patients with grade 1, grade 2, or grade 3 squamous cells carcinoma of the larynx

**Fig. 4:**
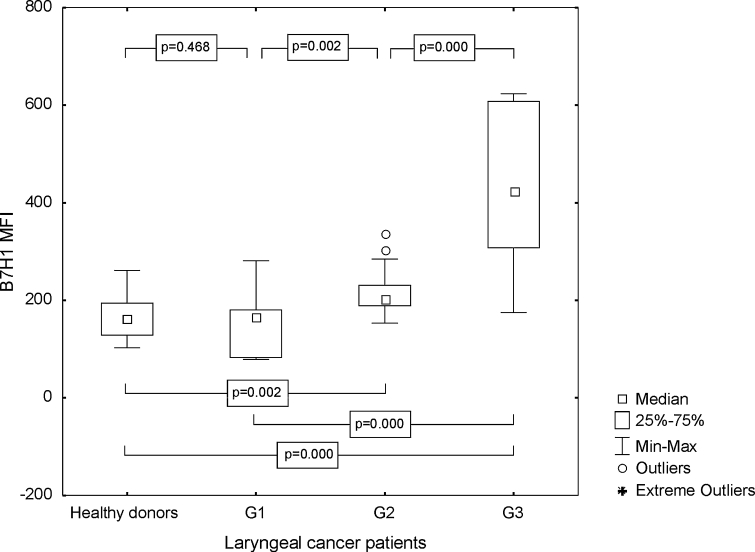
Assessment of the B7H1 MFI values on Mo-DC in control group and laryngeal cancer patients with grade 1, grade 2, or grade 3 squamous cells carcinoma of the larynx

It has been demonstrated that the percentage of Mo-DC, stimulated with LC antigens, characterized by B7H1 expression, derived from the group of patients was significantly statistically higher than the frequencies of CD83+ B7H1+ Mo-DC generated from the control group (median 87.81 %, range from 42.13 to 99.67 %; mean 79.26 ± 18.74 vs. 74.66 %, range from 42.95 to 94.72 %; mean 71.27 ± 15.71 %, *p* = 0.041). Obtained results were the most prominent in G3 LC patients (Fig. 3). The MFI of B7H1 was also higher in patients than in HD (median 209.38, range from 78.84 to 623.68; mean 273.21 ± 151.50 vs. median 163.82, range from 102.93 to 261.15; mean 164.83 ± 46.07, *p* = 0.002). Differences between B7H1 MFI on Mo-DC generated from patients in G1, G2, G3, and from control group are presented in Fig. 4.

**Fig. 5:**
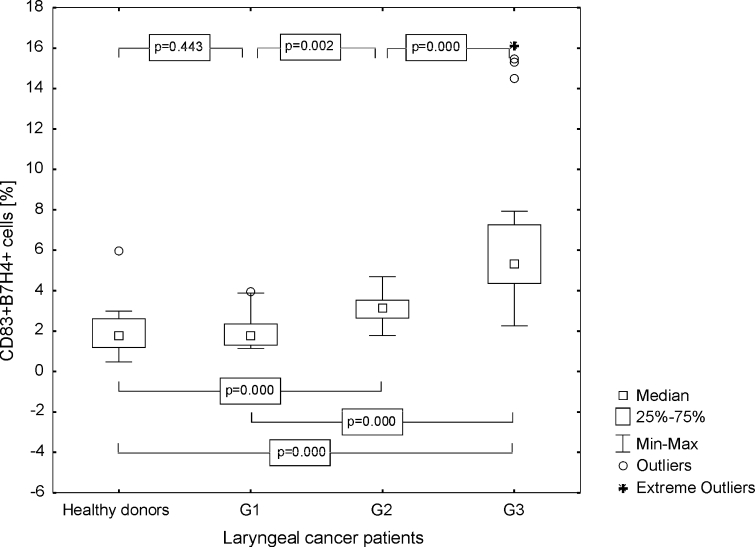
Assessment of the frequencies of CD83+ B7H4+ Mo-DC in control group and laryngeal cancer patients with grade 1, grade 2, or grade 3 squamous cells carcinoma of the larynx

**Fig. 6:**
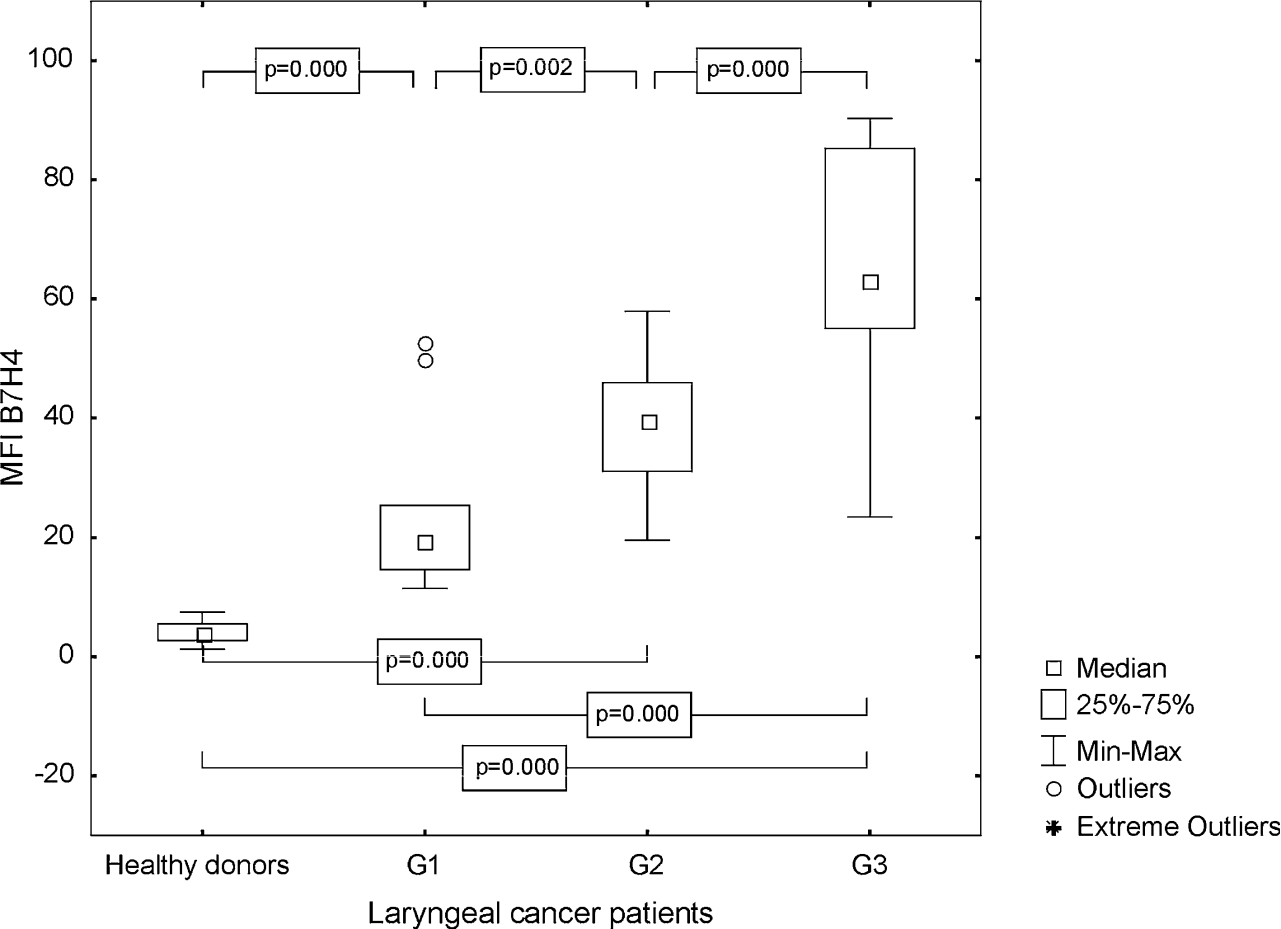
Assessment of the B7H4 MFI values on Mo-DC in control group and laryngeal cancer patients with grade 1, grade 2, or grade 3 squamous cells carcinoma of the larynx

The percentage of mature DC with B7H4 expression was also higher in the tested group than in the control group (median 3.41 %, range from 1.14 to 16.16 %; mean 4.24 ± 3.38 vs. 1.82 %, range from 0.47 to 5.97 %; mean 1.96 ± 1.34 %, *p* ≤ 0.0001). Differences between Mo-DC generated from G1, G2, and G3 LC specimens were significant. The highest frequencies of B7H4+ Mo-DC were found in poorly differentiated LC (Fig. 5). The obtained MFI for B7H4 in the group of the individuals with LC was as follows, median 42.99, range from 11.44 to 90.30; mean 44.76 ± 21.91, and statistically significantly higher than in HD (median 4.02, range from 1.23 to 7.43; mean 4.11 ± 1.69, *p* ≤ 0.0001), especially in the case of G3 LC (Fig. 6).

**Fig. 7:**
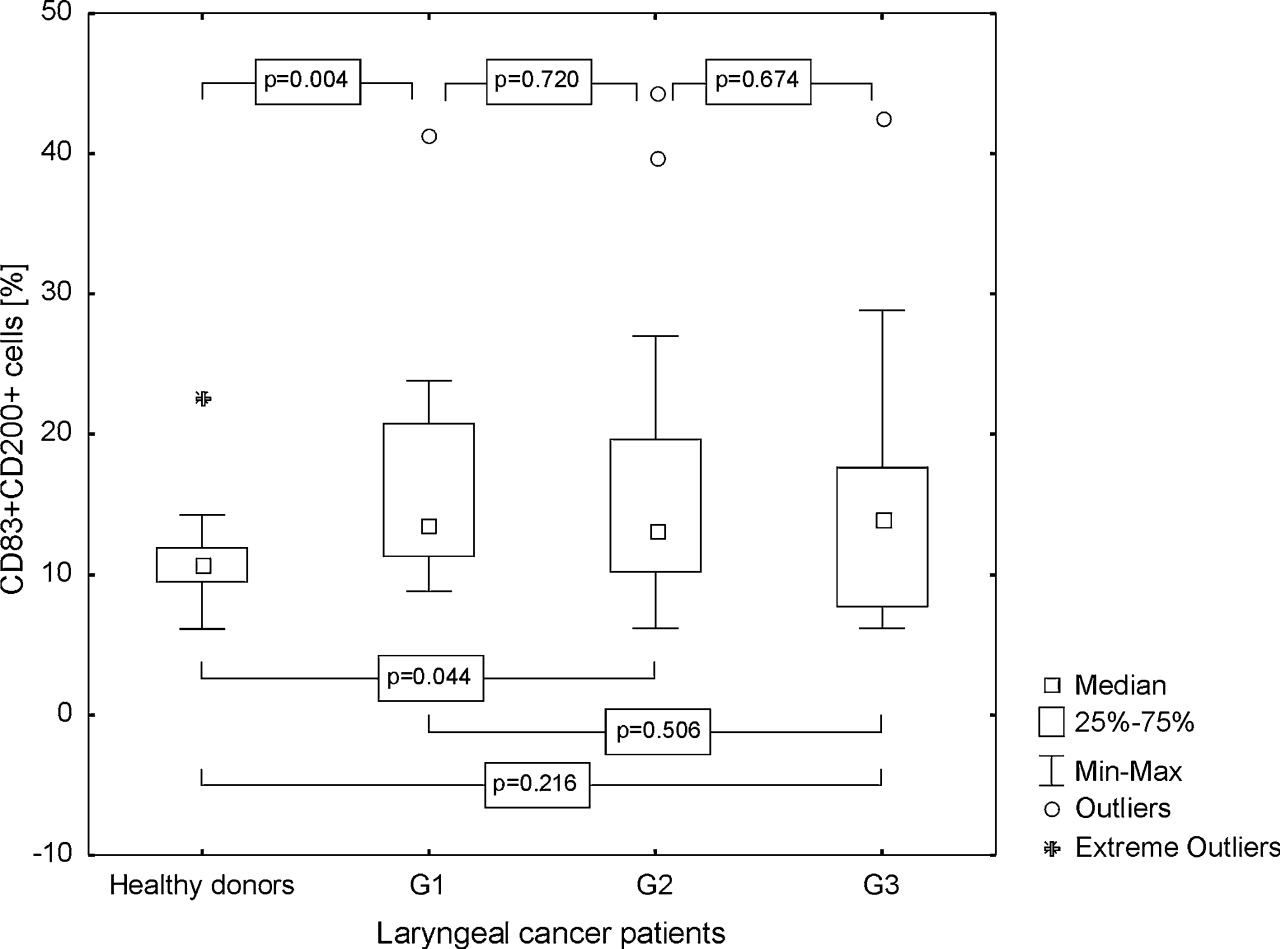
Assessment of the frequencies of CD83+ CD200+ Mo-DC in control group and laryngeal cancer patients with grade 1, grade 2, or grade 3 squamous cells carcinoma of the larynx

**Fig. 8:**
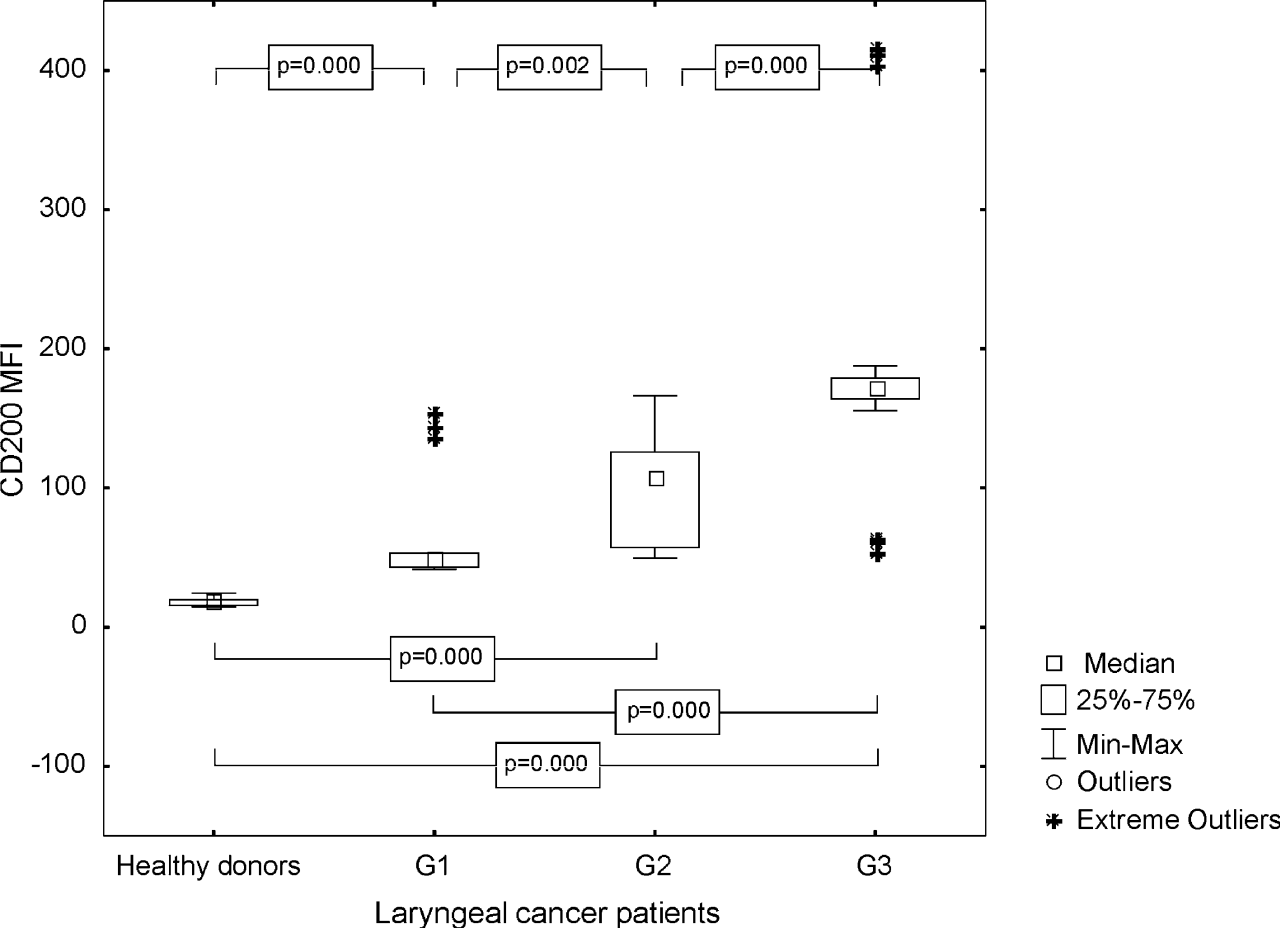
Assessment of the CD200 MFI values on Mo-DC in control group and laryngeal cancer patients with grade 1, grade 2, or grade 3 squamous cells carcinoma of the larynx

The proportion of CD83+ CD200+ cells in patients was higher than in the healthy persons (median 13.71 %, range from 6.17 to 44.47 %; mean 15.99 ± 8.99% vs. median 10.79 %, range from 6.12 to 22.68 %; mean 11.22 ± 3.85 %, *p* = 0.02), and the highest frequencies of the described cells were noted in G3 LC (Fig. 7). Median MFI value of CD200 antigen on the CD83+ Mo-DC from laryngeal cancer patients, stimulated with aTCL, amounted to 109.82 (range from 41.56 to 415.09; mean 120.97 ± 84.68) and was statistically significantly higher than on the unpulsed Mo-DC of HDs (median 18.25, range from 14.41 to 24.47; mean 18.16 ± 2.83, *p* ≤ 0.0001). In patients suffering from poorly differentiated LC, the highest MFI of CD200 molecule was noted (Fig. 8).

**Fig. 9:**
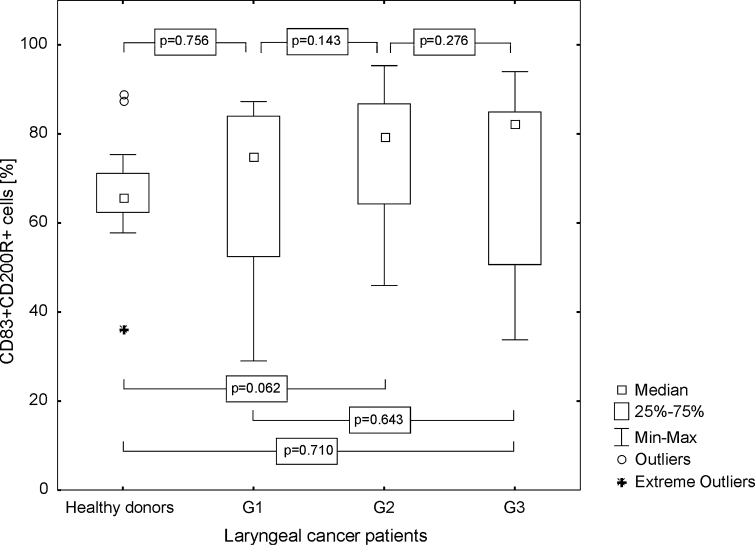
Assessment of the frequencies of CD83+ CD200R+ Mo-DC in control group and laryngeal cancer patients with grade 1, grade 2, or grade 3 squamous cells carcinoma of the larynx

**Fig. 10:**
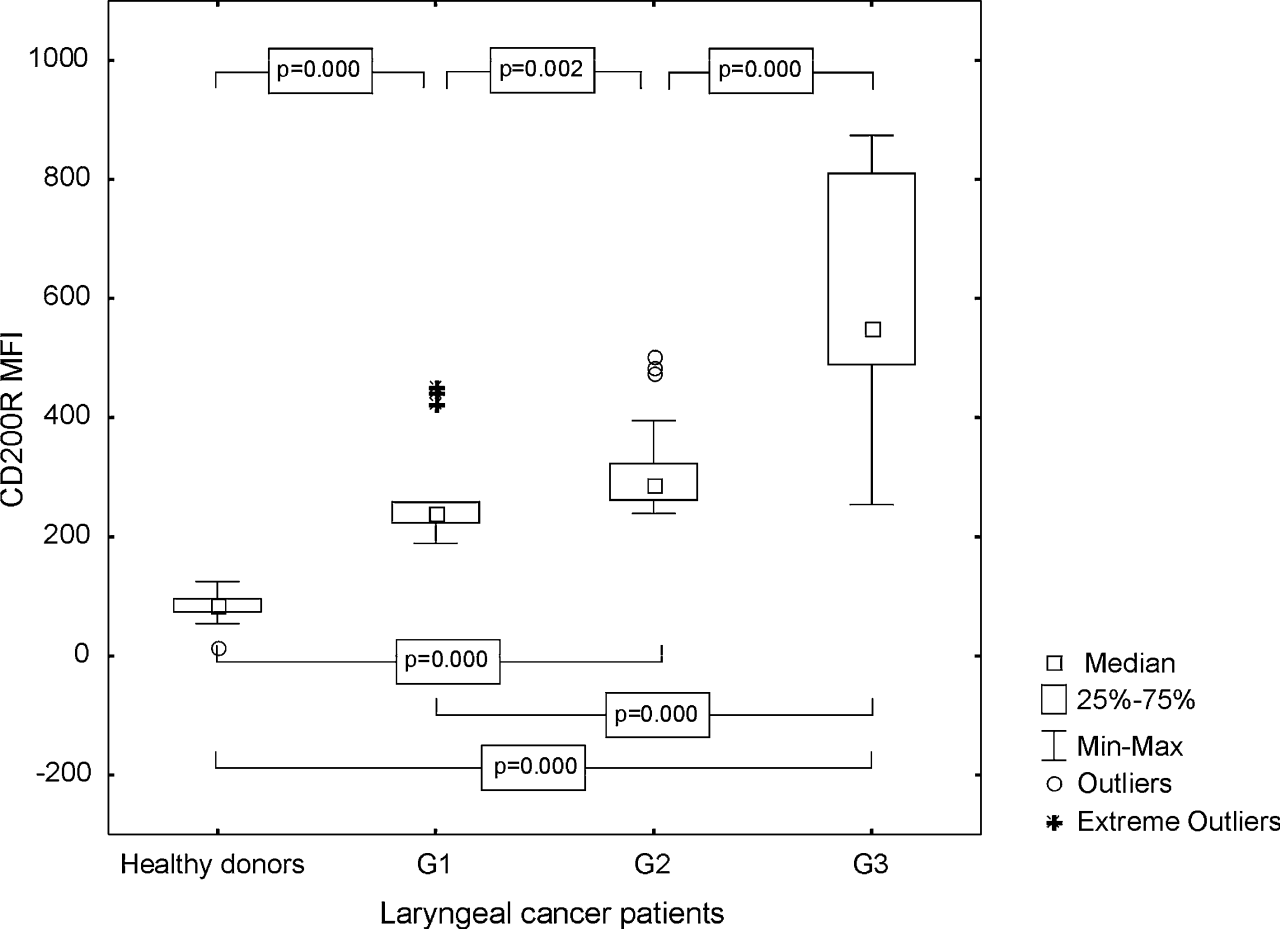
Assessment of the CD200R MFI values on Mo-DC in control group and laryngeal cancer patients with grade 1, grade 2, or grade 3 squamous cells carcinoma of the larynx

The control group was characterized by a lower percentage value of CD83+ Mo-DC with CD200R antigen expression (median 65.87 %, range from 36.21 to 89.05 %; mean 65.78 ± 12.38 %) than individuals with laryngeal cancer (median 79.86 %, range from 29.03 to 95.34 %; mean 72.01 ± 16.98 %), but the statistical significance was also not revealed (*p* = 0.277, Fig. 9). MFI of CD200 receptor expression on CD83+ Mo-DC among the persons with LC was considerably higher (mean 411.91 ± 205.74, median 303.25, range from 188.63 to 874.18) than in the control group (mean 81.62 ± 24.94, median 86.73, range from 16.99 to 124.78, *p* ≤ 0.0001) and the highest values ware noted in G3 LC (Fig. 10).

## Discussion

Patients with advanced cancer including squamous cell carcinoma of the head and neck are known to be immunologically compromised [13, 14]. Tumor cells can therefore escape from the immune responses by numerous mechanisms, including the molecule B7H1, B7H4, and CD200 that acts as a potent suppressor of CD200R-expressing immune cells [15–17].

B7 molecules on the surface of antigen presenting cells participate in the most important activation cycle in the process of co-stimulation which constitutes a ‘second signal’ and is essential to activate T lymphocytes properly by means of an interaction with CD28 molecule on T lymphocytes [18]. B7H1 demonstrates a strong expression of activated DC, T lymphocytes, B lymphocytes, and monocytes [19]. Allegedly, B7H1 molecule, which is present on DC, influences the hindrance of T lymphocyte proliferation dependent on TCR and the decrease in cytokine production [20]. It can be significant while regulating the immune response and the tolerance induction. In our research, it has been observed statistically significantly higher percentage of CD83+ B7H1+ cells in the group of the individuals with LC. The tests conducted on an animal model [21] demonstrated that the obstruction of B7H1 molecule will result in the increase in interleukin 2 and interferon *γ* production. Our results suggest that Mo-DC of the LC patients demonstrate a hindering influence on the T lymphocyte activation and proliferation by means of B7H1 molecule, which is overexpressed especially in LC grade 3.

B7H4 molecule, which interacts with its receptor, also blocks the activation of T lymphocytes, the production of cytokines and it limits cytotoxicity. The role of B7H4 is also connected with the prevention of the apoptosis of cancer cells. It has been demonstrated that interleukin 10 increases the expression of B7H4 on the surface of human cells which presents an antigen and the factor which stimulates the colonies of granulocytes and macrophages and interleukin 4 decreases the expression of this molecule [22]. Our study indicates that there is an overexpression of B7H4 molecule on DC generated from patients with LC and it corresponds with histological grade of LC.

Expression of CD200 has been implicated in multiple types of human cancer including squamous cell carcinoma. However, the impact of tumor expression of CD200 on tumor immunity remains poorly understood and the expression of the discussed molecules was not assessed on the Mo-DC of LC patients. CD200 molecule or its natural ligand plays role in the delivery of a tolerizing signal. Our study revealed that there is a significant relationship between the presence and grade of LC, and the expression of CD200 and CD200R molecules on the Mo-DC pulsed with autologous cancer cell lysates. CD83+ Mo-DC of healthy controls present significantly lower MFI values of CD200 and CD200R antigen expression than the same type of cells after the stimulation. This finding means that pulsing DC with laryngeal cancer tumor lysates would increase the expression of CD200s known inhibitory effect on myeloid cells and is quite surprising. Many studies published before [23, 24] used the concept of pulsing DC with autologous tumor lysates to elicit an effective antitumor immune response. CD200 positive cells can inhibit the stimulation of type-1 cytokine production by bone-marrow-derived B7-1 (and B7-2) positive DC [25]. Other data have implied an immunoregulatory role for CD200 expression, assayed by altered cytokine production in vitro from cells stimulated in the presence or absence of expressed CD200 [26], which is nowadays widely accepted as immunosuppressive factor. Hoek et al. [27] suggested that binding of CD200 antigen with its specific receptor CD200R on myeloid cells such as macrophages, is responsible for down regulation of its activity, so the tissue damage caused by macrophages might be naturally reduced. Rosenblum et al. [28] marked that higher expressions of CD200 were on DC that undergo apoptosis. The most recent study, provided by Seeds et al. [29] shows that CD200 knock-out macrophages produce more IFNα than wild-type macrophages in response to stimulus, what was consistent with CD200s known inhibitory effect on myeloid cells. In contrast, blocking CD200 with an anti-CD200 mAb resulted in reduced IFNα production. This suggests there could be a differential effect of CD200 on IFN induction pathways in DC and macrophages. Moreover, authors’ results support the hypothesis that CD200 is involved in virally induced type I IFN induction.

A number of mAbs have been successfully used in solid cancers. Because CD200 does not have a signaling domain it appears that anti-CD200 Abs do not directly affect targeted tumor cells, and Kretz-Rommel et al. [30] demonstrated that the anti-CD200 mAbs, as expected, did not inhibit tumor cell proliferation or directly induce cell death [31]. Therefore, antagonistic anti-CD200 Abs are expected to exert their effect by blocking immune suppression. Our data indicate that also DC-based immunotherapy supported by the use of mAb anti-CD200 may be effective in the treatment of laryngeal cancer in future, but further studies analyzing the influence of both mAb anti-CD200 and anti-CD200R, and Mo-DC on autologous laryngeal cancer cell cultures are essential.

## Conclusions

Our results prove that there is a relation between the presence of laryngeal cancer and the expression of B7H1, B7H4, CD200, and CD200R regulatory molecules on the CD83+ Mo-DC pulsed with autologous cancer cell lysates. Strong association of LC grade and the tested antigens expression suggests a critical role for these proteins in LC biology. The expression of the aforementioned tolerogenic molecules on mature dendritic cells can be responsible for the failure of the immunotherapy of squamous cell carcinoma of the larynx with the use of DC, which have been stimulated with autologous cancer cells lysates. The implementation of the B7H1, B7H4, and CD200 inhibitors to DC cancer vaccines seems to be appropriate, and could make the immune system of a host more sensitive to cancer antigens by means of unblocking the hindering influence of the mentioned molecules on the cytotoxicity of T lymphocytes.
